# Does active leisure participation promote psychological capital through peer support in economically disadvantaged children?

**DOI:** 10.1371/journal.pone.0234143

**Published:** 2020-06-01

**Authors:** Hung-Ming Tu

**Affiliations:** Department of Horticulture, National Chung Hsing University, Taichung, Taiwan; The Open University, UNITED KINGDOM

## Abstract

This study’s main purpose was to explore the effects of active leisure participation on poor children’s psychological capital, while also investigating the mediated effect of peer support. The sample consisted of 483 economically disadvantaged children, selected and analysed from the Taiwan Database of Children and Youth in Poverty (fifth wave). The study employed partial least squares-structural equation modelling to analyse the relationship between the variables: active leisure participation (exercise and nature travel), peer support, and psychological capital in economically disadvantaged children. The results showed that active leisure participation improved psychological capital and peer support in economically disadvantaged students; and peer support was an important mediator between the other two variables.

## Introduction

The concept of poverty, as Ravallion explained, involves attaining the absolute minimum for survival based on society’s standards [1]. For example, the international poverty line was US$1.90 a day in 2015 [2]. Different countries have contrasting poverty standards [1] and although it is impossible to establish an absolute poverty line for all countries, most governments have specific criteria to define economically disadvantaged households, such as total income, unemployment, disability, chronic illness, and single parenthood [3]. The Taiwanese government initiated the Public Assistance Act to define low-income standards in accordance with the lower than minimum living index determined by county governments. The Taiwan Fund for Children and Families (TFCF) is an international non-governmental organisation that offers assistance to economically disadvantaged families. It defines the latter as the inability to attain the bare minimum for living and includes households containing children below the age of 18, one or both deceased parents, or one parent who loses the ability to work [4,5]. This TFCF definition also covers local minimum living needs and potential economically disadvantaged conditions. For the purpose of this study, this is the chosen definition for determining the participants’ economical condition.

The challenges hindering childhood development under poverty conditions are important issues that should be paid careful attention [6]. People subjected to poverty are often impacted by long-term negative experiences throughout their lives [6,7]. Poverty conditions affect children’s psychological health by causing emotional problems, depression, shyness, and lower expressive and attentional abilities [8–10]. Therefore, finding useful methods to improve or reduce long-term negative experiences has become an important objective in child development and future growth.

Positive psychology helps disadvantaged people pursue self-actualising vocations [7]. It also facilitates social and psychological developments, helps them overcome adversity [11–13], and provides cognitive strategies that build a sense of self-efficacy, optimism, hope, and resilience, which are all comprised in the term psychological capital [7,14]. These four positive psychological states of development involve these personal expressions: (1) self-efficacy: having enough confidence to successfully achieve task; (2) optimism: positively thinking about the causes of life and future events; (3) hope: having a positive and motivated state regarding success and the future; and (4) resiliency: developing the capacity to rebound from negative events to achieve success [7,14]. These factors are also important in helping children overcome adversity [11–13]. Some studies have indicated that leisure participation is associated with psychological capital. Stewart, Smith, and Moroney, for example, found that gym exercises strengthened self-esteem, confidence, and psychological resilience [15]. Promoting positive psychology through leisure participation may be a useful strategy for child development, especially for those living in poverty. Therefore, this study explored the relationship between leisure participation and psychological capital in children from economically disadvantaged families.

### Active leisure participation and psychological capital

Active leisure participation contributes to positive psychology [15] and active lifestyles [16]. Active leisure participation involves physical activity, such as regular exercise, sports activities, gym workouts, cycling, swimming, and mountain climbing [17,18]. Past studies have shown that active leisure participation is positively associated with psychological well-being among adolescents [17,18]. Other studies have also indicated that this form of participation, combined with experiencing nature, is highly appealing [19] and benefits health [20]. Experiences in nature facilitate attention restoration, reduce stress levels, and produce positive emotions [21]. In the study of Salvini et al., higher physical activity was positively linked to psychological well-being among schoolchildren from disadvantaged neighbourhoods [22]. Breslin et al. also indicated that physical activity had a positive relationship with well-being for socially deprived children [23]. Further, Crews et al. noted that participating in aerobic exercise positively improved psychological well-being among children in low-income districts [24]. Therefore, active leisure participation may be a method of facilitating children’s positive psychology. This study hypothesised that the former affects the psychological capital of economically disadvantaged children (Hypothesis 1).

### Role of peer support in active leisure participation and psychological capital

Poverty produces leisure constraints in terms of spending [25] and time availability [26], which results in low leisure participation. In a Save the Children UK survey, children in severe poverty were shown to have lower leisure opportunities than both non-poor and non-severely poor children [27]. Children can easily lose their social circle and become socially isolated, which in turn induces social exclusion and results in more poverty related negative experiences, because of their inability to participate in leisure activities [26,28].

Social relationships are critical for growth and child development [29]. Low-income families often experience economic pressure that increases the probability of children being rejected by their peers [30], as they have fewer resources to engage in peer and school activities, forcing them to face the challenge of reduced social interactions and peer support [31]. Therefore, poverty is a negative life experience that constrains the establishment of peer support through low leisure participation. Promoting the latter may be a useful method to improve poor children’s social relationships. Leisure participation is a positive factor in a child’s social life [21,32], because his/her engagement in these activities produces social connections [33]. Therefore, active leisure participation can perhaps be a means of facilitating a child’s social relationships.

Social relationships form important connections and fulfil basic psychological needs for maintaining intrinsic motivation [34,35]. Some leisure studies have indicated that social support operates as an important linking mechanism between leisure activities and psychological development [35–38]. It is also an important mechanism between leisure participation and negative emotion, as this participation buffers the effects of psychological pressure on physical and mental health [35,38]. In child development, play behaviour produces social resilience through the process of cooperation and negotiation [39]. Children in poverty may have restricted leisure participation which affects their social and emotional development [39]. Promoting children’s leisure participation can improve social support and child development despite their economic situation. Therefore, this study hypothesised that active leisure participation affects peer support in economically disadvantaged children (Hypothesis 2) and peer support also affects psychological capital in that target population (Hypothesis 3).

### Purpose of the study

**Fig 1:**
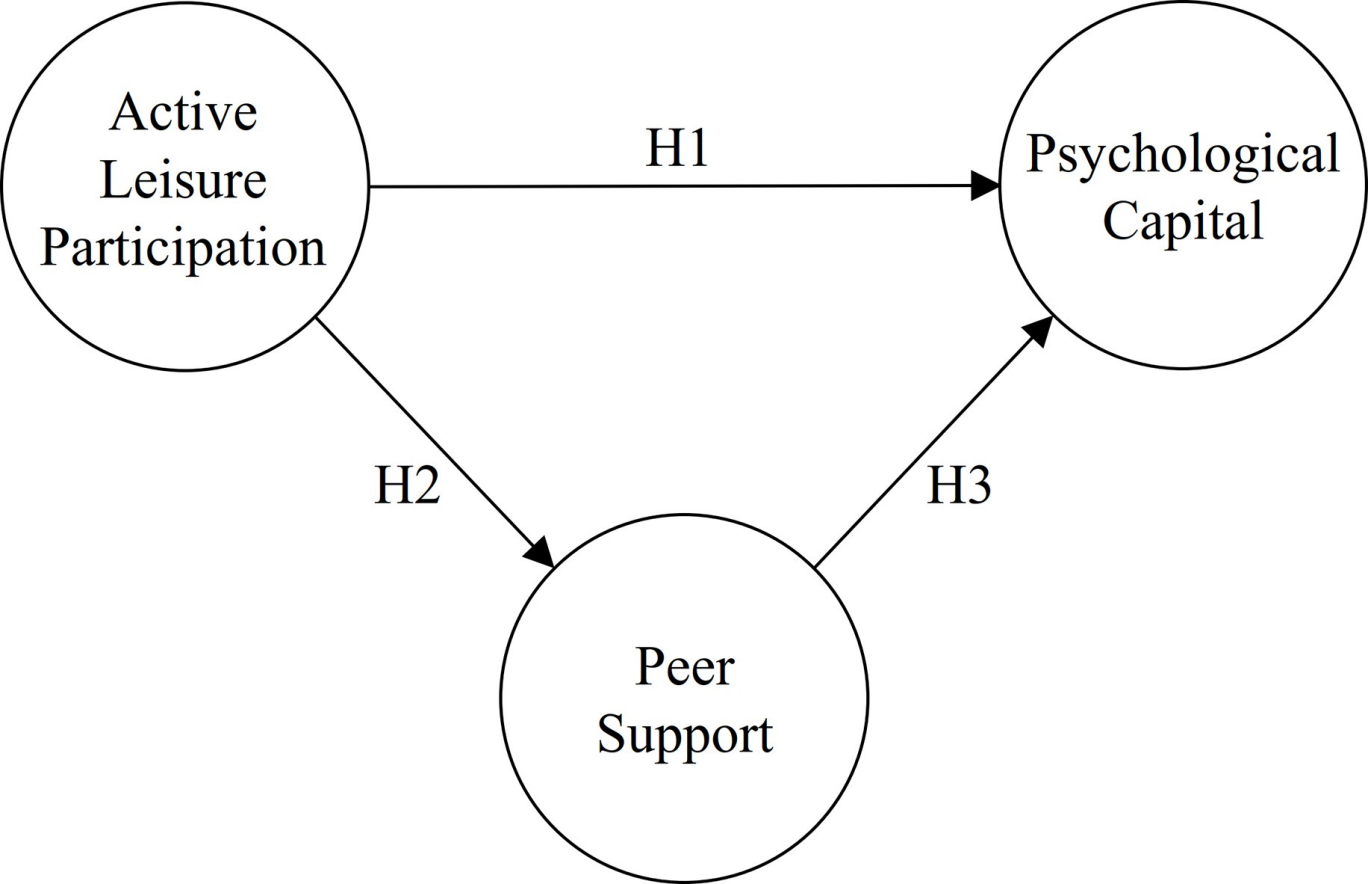
Research framework.

Poverty is a powerful constraint on leisure participation [40,41]. One of the reasons for this is that people cannot afford the costs involved in seeking leisure and recreation [41,42]. Free attractions are an important potential option although they lack the same depth or range of experiences as those that require fees [41]. Thus, as poverty often produces a shortage of recreational and leisure resources, some references suggest increasing leisure opportunities for children in poverty [27]. Understanding the effects of leisure participation on the psychological capital of poor children is an important step in providing evidence, while deciding on subsidy policies. Health-related behaviours differ across socioeconomic groups [43], thus, leisure participation’s effect on children’s psychological capital should be examined. However, few studies have produced scholarly evidence on the benefits of this effect on poor children. To fill the gaps in existing research, the main purpose of this study is to explore the effects of active leisure participation on the psychological capital of economically disadvantaged children. This study also seeks to enhance the understanding of the ways reduced leisure participation affects the psychological capital of the target population. Peer support may be an important interacting mechanism between leisure participation and poor children’s psychological capital. Therefore, the second purpose of this study is to explore the mediated effect of peer support on the two aforementioned factors (Fig 1).

## Method

### Study population

The secondary data for this study stems from the Taiwan Database of Children and Youth in Poverty (TDCYP), a long-term survey conducted by the TFCF [5]. The TFCF branches covering Taiwanese cities collected the data to capture poverty dynamics and potential risk factors for child development through long-term follow-ups. The study’s population comprised economically disadvantaged families supported through TFCF assistance. The TDCYP contained five waves: wave 1 in 2009, wave 2 in 2011, wave 3 in 2013, wave 4 in 2015, and wave 5 in 2017. The first four waves sampled children through a systematic random sampling of 42,167 economically disadvantaged children on November 12, 2008. The fifth wave selected 2,303 people from the four previous waves’ follow-up samples. They collected data from 1 July 2017 to 31 December 2017 and circulated an electronic questionnaire through multiple survey methods, including group tests in the TFCF branches, individual interviews, and physical and electronic mail. The TDCYP data has been implemented irreversible anonymisation to provide the analysis of academic research and are available from the Taiwan’s Survey Research Data Archive (Doi:10.6141/TW-SRDA-D00166-1). A total of 1,618 people completed the questionnaire, including 483 children who were 13–18 years. The survey response rate was 70.3%. Considering the importance of the latest data and the critical variables of child positive psychology, peer support, and the effects of age differences, this study selected and analysed the 483 children from the fifth wave.

The study sample included 483 children: 217 participants were boys (44.9%) (Table 1). Regarding age differences, 75 participants (15.5%) were between 13 and 15 years old, and 408 between 16 and 18 (84.5%). In terms of poverty type, 275 (56.9%) and 79 (16.4%) participants were from low-income households and middle-to-low-income households, respectively, based on their local government’s affirmation. A total of 129 participants (26.7%) were from economically disadvantaged families, according to the TFCF definition. Additionally, 54 (11.2%), 48 (9.9%), 277 (57.3%), and 104 (21.5%) of the participants corresponded to the no-parent, single-parent father, single-parent mother, and two-parent households, respectively.

**Table 1:** Descriptive statistics (N = 483).

Variables	N (%)	
Gender		
Boy	217	(44.9)
Girl	266	(55.1)
Age		
13–15 years	75	(15.5)
16–18 years	408	(84.5)
Type of poverty		
Low-income household	275	(56.9)
Middle-to-low-income household	79	(16.4)
Economically disadvantaged family	129	(26.7)
Type of family		
No-parent	54	(11.2)
Single-parent father	48	(9.9)
Single-parent mother	277	(57.3)
Two-parent	104	(21.5)
Active leisure participation (original)		
Participate exercise frequently	254	(52.6)
Participate nature travel frequently	40	(8.3)
Active leisure participation (dummy variable)		
Participate exercise and nature travel infrequently (code 0)	217	(44.9)
Participate exercise or nature travel frequently (code 1)	266	(55.1)

### Measures

#### Active leisure participation

The independent variable in this study was active leisure participation. This study extracted data on participation in exercise and nature travel to assess the independent variable using a multiple-choice question format. Exercise included sports, swimming, dance, fitness, etc. Nature travel involved mountain hiking, travel, fishing, shrimp fishing, etc. As leisure was not a major issue in the TDCYP survey, the original questionnaire used a binary variable to survey leisure participation: ‘participate infrequently’ or ‘participate frequently’.

#### Peer support

In the TDCYP survey, Chiang and Chen extracted three items to assess peer support for economically disadvantaged children: ‘I can share my feelings with my classmates’; ‘my classmates and I can understand each other’; and ‘I can share my life and heart with my classmates’ [44]. A four-point scale, with good reliability (Cronbach’s alpha = 0.93) [44]^,^ rated their degree of agreement from 1 (completely disagree) to 4 (completely agree).

#### Psychological capital

Psychological capital contained four elements: self-efficacy, hope, optimism, and resilience [7,14,44]. In indigenous studies, Chiang and Chen extracted these four items from the TDCYP survey to assess psychological capital [44], according to the definitions of Brooks & Goldstein [45], Luthans [46], Luthans et al. [47], and Luthans et al. [14]. Self-efficacy was ‘I energetically plan something’ which represents children having the confidence to face challenges. Optimism was ‘I work hard to get success’ which signifies children thinking positively about achieving success. Hope was ‘I feel confident about the future’ which represents children being positively motivated for the future. Resiliency was ‘I plan to improve future living standards’ which constitutes children having the capacity to rebound from poverty to improve their lives. This scale, as the previous one by the same authors, also had good reliability (Cronbach’s alpha = 0.86) [44]. This scale had also established criterion-related validity on academic performance [44], indicating that the four psychological items can measure a child’s psychological capital. Therefore, the present study employed these four elements to rate the degree of psychological capital, during its three month span, using a five-point scale from 1 (seldom) to 5 (always).

### Data analysis

The study performed the Kolmogorove-Smirnov and Shapiro-Wilk normality tests with SPSS version 22, and used skewness and kurtosis values to test the data’s normality. The variables of peer support and psychological capital presented non-normality. PLS-SEM is a useful method to handle the problematic modelling of non-normal data [48–50]. In the analysis of active leisure participation, this study combined exercise and nature travel items, and recorded them into a single dummy variable: ‘participate in exercise and nature travel infrequently’ (code 0) and ‘participate in exercise or nature travel frequently’ (code 1) because only one-tenth of children participated nature travel frequently (10.1%). PLS-SEM also work well with the single-item variable and binary data [48–50] to handle the single-item binary variable of active leisure participation. Therefore, PLS-SEM analysed the relationship between active leisure participation and child psychological capital in economically disadvantaged children. SmartPLS 3 [51] software served its purpose in all analysis processes.

The study also employed the reflective measurement model shown below for all evaluations: (1) cronbach’s alpha and composite reliability (CR) should be higher than 0.70 to indicate high internal consistency; (2) outer loadings should be higher than 0.70 to signify high indicator reliability in convergent validity; (3) the average variance extracted (AVE) should be higher than 0.50 to demonstrate sufficient convergent validity; (4) the indicator’s outer loading should be higher than other constructs’ cross-loadings to signify discriminant validity; and (5) AVE’s square root should be higher than other constructs’ correlations (Fornell-Larker criterion) [48].

The structural model evaluation followed these steps: (1) noting that a predictor’s variance inflation faction (VIF) should be higher than 0.20 and lower than 5.0 to test collinearity; (2) using 5,000 bootstrapped samples to assess the statistical significance of path coefficients; (3) evaluating in-sample predictive power through *R*^2^ value (0.25, 0.50, and 0.75 are weak, moderate, and large, respectively); (4) evaluating effect size *f*^2^ value (0.02, 0.15, and 0.35 are small, medium, and large effects, respectively); (5) evaluating out-of-sample predictive power through Q^2^ values (Q^2^ should be larger than zero); and (6) evaluating effect size *q*^2^ value (0.02, 0.15, and 0.35 are small, medium, and large predictive relevances, respectively) [48].

## Results

Regarding active leisure participation, 52.6% frequently participated in exercises, while 8.3% frequently engaged in nature travel (Table 1). In the dummy variable of active leisure participation, 44.9% participated in exercise and nature travel infrequently, and 55.1% frequently engaged in these activities. All peer support and psychological capital items’ mean scores were higher than 2.99 (Table 2).

**Table 2:** Quality criteria: Factor loading, cross loading, discriminant validity, construct reliability and validity.

Variables	M (SD)		Factor loading and cross loading			Cronbach’s alpha	Composite Reliability (CR)	Average Variance Extracted (AVE)
			A	C	P			
Leisure participation						1.00	1.00	1.00
A1: Active leisure	0.55	(0.50)	1.00	0.12	0.12			
Peer support						0.93	0.95	0.87
C1: share my feelings	3.06	(0.62)	0.10	0.92	0.32			
C2: understand each other	2.99	(0.65)	0.12	0.94	0.40			
C3: share my life and heart	3.00	(0.66)	0.09	0.94	0.37			
Psychological capital						0.88	0.92	0.73
P1: Hope	3.19	(0.91)	0.13	0.39	0.84			
P2: Optimism	3.26	(0.93)	0.09	0.31	0.85			
P3: Resiliency	3.30	(0.92)	0.10	0.31	0.86			
P4: Self-efficacy	3.28	(0.89)	0.08	0.31	0.88			

All cronbach’s alpha and CR values were higher than 0.70, indicating high internal consistency (Table 2). All AVE values were higher than 0.50, demonstrating a sufficient convergent validity. All outer loadings were also higher than 0.70, showing high indicator reliability in convergent validity. The indicator’s outer loadings were higher than other constructs’ cross-loadings. AVE’s square root was also higher than other constructs’ correlations, designating discriminant validity (Table 3).

**Table 3:** Fornell-Larker criterion: The square root of AVE should be higher than other construct’s correlation.

	Active leisure participation	Peer support	Psychological capital
Active leisure participation	1.00		
Peer support	0.12	0.94	
Psychological capital	0.12	0.39	0.86

**Fig 2:**
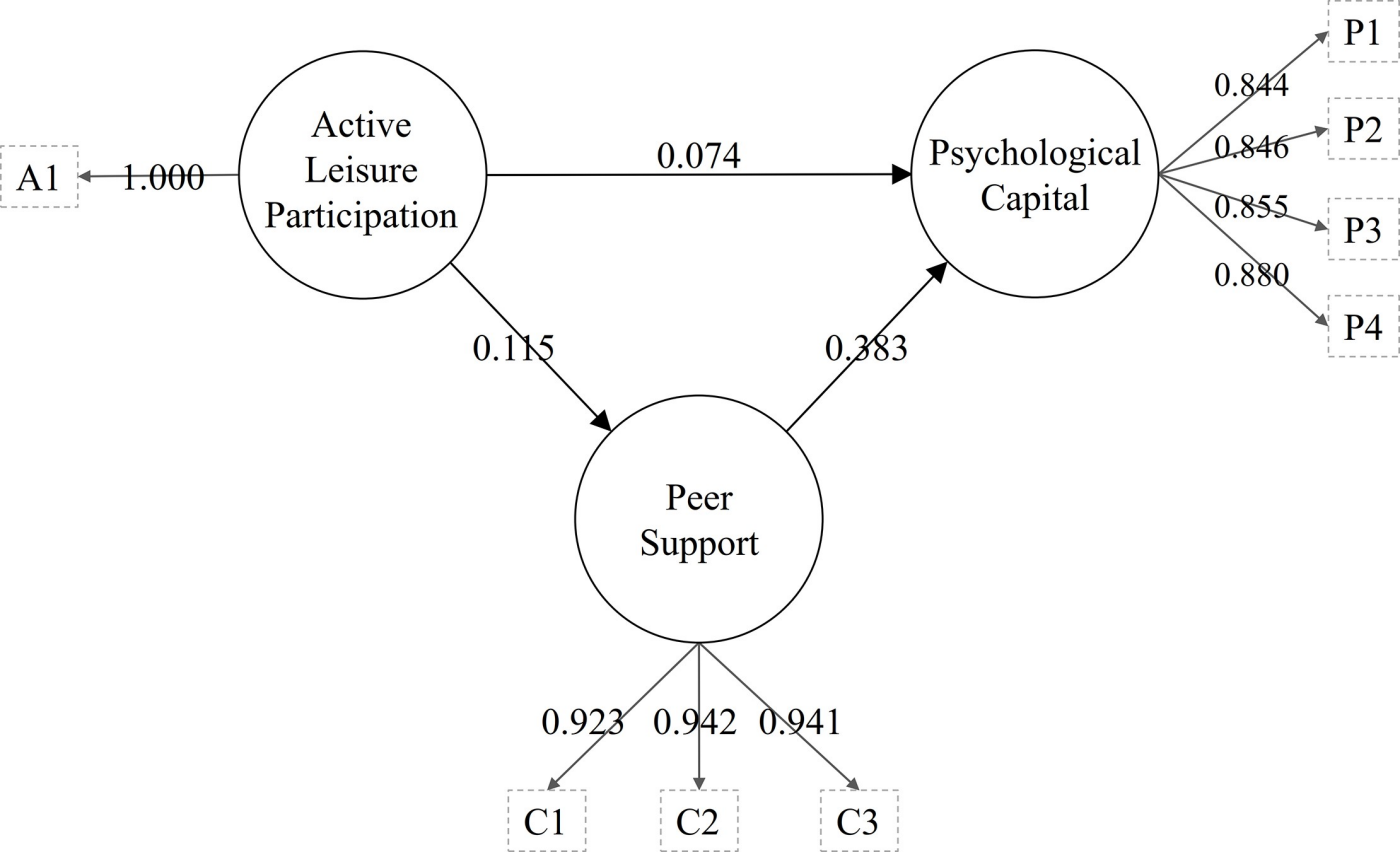
The analysis of PLS-SEM among active leisure participation, classmate support, and psychological capital.

All indicators’ VIFs were higher than 0.20 and lower than 5.0. This suggests the absence of collinearity in this study. Table 4 and Fig 2 present the PLS analysis results. First, active leisure participation had a positive and small total effect on psychological capital (*β* = .12, BC 95% CI of .03 to .21, *f*^2^ = .01), supporting Hypothesis 1. Second, the former also had a positive and small direct effect on peer support (*β* = .12, BC 95% CI of .03 to .20, *f*^2^ = .02), supporting Hypothesis 2. Third, peer support had a positive and medium direct effect on psychological capital (*β* = .39, BC 95% CI of .28 to .47, *f*^2^ = .17), supporting Hypothesis 3.

**Table 4:** Path coefficients of direct effects and indirect effects.

Parameter	Coefficient	T value	BC 95% CI	
Active leisure participation → Psychological capital (Total effect)	.12**	2.68	.03,	.21
Active leisure participation → Psychological capital (Indirect effect)	.04**	2.44	.01,	.08
Active leisure participation → Psychological capital (Direct effect)	.08	1.77	-.01,	.16
Active leisure participation → Peer support (Direct effect)	.12**	2.60	.03,	.20
Peer support → Psychological capital (Direct effect)	.39***	7.80	.28,	.47

*p < 0.05

**p < 0.01

***p < 0.01

BC CI: Bias-corrected confidence interval based on 5,000 bootstrapped samples.

The results additionally show that the in-sample’s predictive power is weak regarding peer support (*R*^2^ = .01) and medium for psychological capital (*R*^2^ = .16). The Q^2^ values were larger than zero, indicating out-of-sample predictive power (Q^2^ was .01 and .11 in peer support and psychological capital, respectively). The *q*^2^ effect sizes were small: active leisure participation and peer support on psychological capital was .003 and .111, respectively. Finally, direct effect analyses showed that active leisure participation did not directly affect psychological capital (*β* = .08, BC 95% CI of -.01 to .16). Thus, peer support fully mediated the relationship between active leisure participation and psychological capital according to the guideline of Zhao, Lynch, & Chen [52].

## Discussion

Active leisure participation had no direct effect on psychological capital because it was fully mediated by peer support. Active leisure participation had a direct effect on peer support, and subsequently, had an indirect effect on psychological capital in economically disadvantaged children. Although this study hypothesised the relationship of variables through a literature review, there were limitations in using cross-sectional data to evidence the relationship. TDCYP surveyed five waves to form longitudinal data of the participants. Only wave 5 of TDCYP simultaneously surveyed leisure participation, peer support, and psychological capital. Therefore, we only used cross-sectional data of wave 5 to demonstrate the relationship due to data limitation. Indeed, a cross-sectional study only shows associations and is limited to explain causality. Future studies should design and survey using a longitudinal study of leisure participation to evidence the causality among leisure participation, peer support, and psychological capital.

This study’s results showed that active leisure participation had an indirect and positive effect on psychological capital among economically disadvantaged children. Exercise and nature travel appear to be essential tools in the attainment of success, and in promoting self-efficacy, optimism, hope, and resiliency. In this study, nearly half of the target population did not exercise or participate in nature travel frequently. Thus, one should consider providing opportunities to exercise in schools and community environments while drafting child and youth welfare and policies—especially for economically disadvantaged children. Although the effect size was small, three reasons explain this outcome: (1) the relationship is really occurring, but you can only discover it through careful study [53,54,55]; (2) the dummy variable’s limitations perhaps affect the effect size of active leisure participation; and (3) mediator variables may exist within the relationship.

This study also demonstrated that active leisure participation had a direct and positive effect on peer support in economically disadvantaged children. Exercise and nature travel were important positive factors for promoting interactions among classmates. In past studies, nature travel was a positive factor for peer support. Seeland, Dübendorfer, and Hansmann, for example, indicated that leisure activities facilitate social interactions among young people in urban forests and public green spaces [56]. Vadala, Bixler, and James explained that wild land childhood play promotes socialisation and showed that nature travel increases peer support in economically disadvantaged children [57]. Tangeland and Aas similarly indicated that nature-based activities promote family interaction [58].

Accordingly, in this study, peer support was an important mediator between leisure participation and psychological capital for the target population. Exercise and nature travel can be used to promote peer support, as the process creates an important connection between classmates and brings about social support that acts as a buffer against negative emotions [35,38]. In comparison, low active leisure participation induced more serious social constraint and the loss of peer support. In this study, exercise was a useful method to create a social connection with classmates which promotes psychological capital. Nature travel also demonstrated the same benefits.

Past studies have indicated that social relationships play an important role between leisure and psychological development [35–38]. Exercise and nature travel should be encouraged in schools to avoid low peer support and facilitate the development of self-efficacy, hope, optimism, and resilience, especially among economically disadvantaged children. Poverty often produces a negative psychological influence that leads to potentially disadvantageous economic behaviours that may prolong the process of rising out of poverty, or even make the escape from poverty impossible [8]. This study suggests that providing leisure resources is essential for the psychological capital of poor children. For example, Allington et al. indicated that supplying self-selected books reduced reading problems for children from low-income families [59]. Therefore, providing resources for exercise and nature travel, such as leisure facilities, knowledge, activities, and fee discounts, promotes the positive development of economically disadvantaged children. The construction or accessibility of leisure spaces is also an important consideration, because parks and recreation facilities promote physically active leisure behaviours [16].

Despite these encouraging results, this study presents a few limitations. The first limitation is the lack of follow-up. As the Taiwan Database of Children and Youth in Poverty (TDCYP) considered the critical variables of leisure, peer support, and psychological capital, this study used its fifth wave for sample collection. Although waves one to five sampled children through systematic random sampling, a loss of follow-up occurred in the fifth wave. This lack of follow-up may have induced bias and affected the results of this study. To rectify this issue, this study used the PLS-SEM (nonparametric method) to analyse the hypothesis. Although this method has several advantages for analysing data with no distributional assumptions, the lack of a global goodness-of-fit measure limits theory testing and confirmation [48]. Future studies should use random sampling to test and confirm the aforementioned relationships through covariance-based structural equation modelling. However, it is difficult to survey economically disadvantaged children for two reasons: (1) governments and relative organisations protect the information of economically disadvantaged families; and (2) the guardian’s informed consent process is hard to implement. Leisure studies on economically disadvantaged families should collaborate with governments and relative organisations to reduce these challenges and promote survey quality.

The leisure participation data structure is the two limitation. TDCYP’s secondary data recorded participation in exercise and nature travel through the categorical variable ‘participate infrequently’ or ‘participate frequently’. Therefore, participation in exercise and nature travel was the dummy variable in the analysis process. Although using dichotomous endogenous variables in PLS-SEM is problematic, they work well with the process of PLS-SEM [60]. Therefore, dichotomous independent variables of leisure participation data can be used in this study. However, it is also difficult to explain the frequency variety’s effect on the dependent variable of child psychological capital. For future studies, using numbers to measure the frequency of active leisure participation is suitable to predict variables and evaluate the effect size for psychological capital and peer support. Another limitation was the combinative leisure type, which overlooked detailed information and reduced analysis possibilities. Exercise, for example, combined sports, swimming, dance, and fitness. Future study should focus on each different active leisure to explore which produces larger effects on psychological capital and peer support, while providing detailed information on drafting child and youth welfare and policies.
